# Neuroendoscopic lavage versus traditional surgical methods for the early management of posthemorrhagic hydrocephalus in neonates

**DOI:** 10.1007/s00381-022-05606-4

**Published:** 2022-07-13

**Authors:** Aleksandre Dvalishvili, Mirza Khinikadze, Giorgi Gegia, Lali Khutsishvili

**Affiliations:** 1 Gudushauri National Medical Centre, New Vision University, Tbilisi, Georgia; 2 Medical School, New Vision University Hospital, Tbilisi, Georgia; 3 Neurosurgery Department, Tbilisi State Medical University Hospital, Tbilisi, Georgia; 4 Tbilisi State Medical University, Tbilisi, Georgia

**Keywords:** Intraventricular hemorrhage, Neonates, Neuroendoscopic lavage, Posthemorrhagic hydrocephalus

## Abstract

**Objective:**

Despite advances observed in neonatal neurosurgery, treatment of posthemorrhagic hydrocephalus (PHH) remains a major challenge. This study aims to observe the outcomes of the application of the neuroendoscopic method for treating early-stage posthemorrhagic hydrocephalus.

**Methods:**

A total of 60 medical cases were studied retrospectively. From 2016–2021, the patients were treated at the neonatal intensive care unit (NICU). As an initial neurosurgical intervention, 19 neonates (A) underwent neuroendoscopic lavage (NEL) of the ventricular system and evacuation of posthemorrhagic debris via ventricular washout. A total of 36 neonates (B) were treated via traditional surgical methods, out of which 24 neonates underwent ventricular reservoir implantation (VAD) and 12 underwent ventriculostomy (EVD). Of the 60 patients, there were 5 neonates (C), who were treated directly by ventriculoperitoneal (VP) shunting after serial ventricular/lumbar punctures. As the inclusion and surgical criteria were significantly different for this group, their data were evaluated separately. Accordingly, these patients were divided into three (A, B, and C) groups.

**Results:**

The gestational age of group A neonates (31 weeks) was slightly higher than the gestational age of group B (29.1 weeks). During their hospitalization, 15 neonates (78.94%) from group A and 26 (83.87%) neonates from group B required shunting. In group B, 5 patients (12.19%) died before the need for shunting occurred. No lethal outcomes were observed in group A, and 9 (25%) patients from group B died during hospitalization. In group A, central nervous system (CNS) infections developed in 3 patients, which is much less than the 18 patients in group B. NEL was found to give better neurological outcomes in patients with intraventricular hemorrhages. Serial ventricular/lumbar puncture can be used as a life-saving manipulation in very unstable patients to temporarily decreasing intracranial pressure. Its frequent use is associated with brain parenchymal damage and poor neurological outcome.

**Conclusion:**

The neuroendoscopic method of treating neonatal posthemorrhagic hydrocephalus is a safe and effective one. Its application reduces the period of patient hospital stay, the incidence of meningitis, and the frequency of development of multiloculated hydrocephalus.

## Introduction

In preterm infants, treatment of posthemorrhagic hydrocephalus resulting from intraventricular hemorrhage is a serious challenge as there is still no standard treatment regimen reflecting the most effective treatment method [1]. Despite the dramatic improvement in the quality of medical care, the incidence of intracerebral hemorrhage in premature infants remains as high as 25–30% and its complications (mainly post-hemorrhagic hydrocephalus) constitute the additional brain-damaging factors that are caused, on the one hand, by the direct impact of high intracranial pressure on nerve structures, and on the other hand, by the additional immune response triggered by blood and its breakdown products, which get into the ventricles and provokes further damage to the nerve cells [2–4]. It is well estimated that infants with PHH have poorer surgical and neurodevelopmental outcomes [4, 5]. At present, the main goals of intraventricular hemorrhage treatment are reduction of the need for a fluid drainage device (shunt) and protection of nerve structures from further progressive damage [6–9]. The authors Matthias Schulz et al. proposed endoscopic irrigation of the ventricular system using Ringer’s solution. The primary results of neuroendoscopic irrigation of ventricles were impressive: the need for shunting, the number of neurosurgical interventions performed as well as the frequency of developing the neuroinfection and postinfection cysts were low [4]. Our research is designed for further exploration of the above-mentioned issue.

## Methods

A total of 60 cases were studied retrospectively. From 2016 to 2021, the patients were treated at the neonatology departments of Academician O. Ghudushauri National Medical Center and Pediatric Private Clinic. As an initial neurosurgical intervention, 19 neonates (A) underwent endoscopic lavage of the ventricular system and evacuation of posthemorrhagic debris via ventricular washout, and 36 neonates (B) were treated via traditional surgical methods, out of which 24 neonates underwent ventricular reservoir implantation and 12 underwent ventriculostomy. Of the 60 patients, there were 5 neonates (C) who were treated directly by VP shunting after serial ventricular/lumbar punctures. Accordingly, these patients were divided into three (A, B, and C) groups.

In all groups, indications of neurosurgical intervention were as follows: (1) increase of head circumference more than 12 mm per week, (2) progressive ventriculomegaly by ultrasonography, and (3) condition of anterior fontanelle and inter-bone sutures. All patients underwent a brain CT scan during the preoperative preparation phase.

Inclusion criteria were IVH documented on ultrasound or CT; progressive dilation of lateral ventricles. The exclusion criteria were congenital neurosurgical malformations like spina bifida encephalocele.

Statistical analysis for safety results was performed using IBM SPSS software. Statistical tools included the chi-square contingency, table A’s *p*-value < 0.05 was considered statistically significant.

### Surgical treatment

**Fig. 1:**
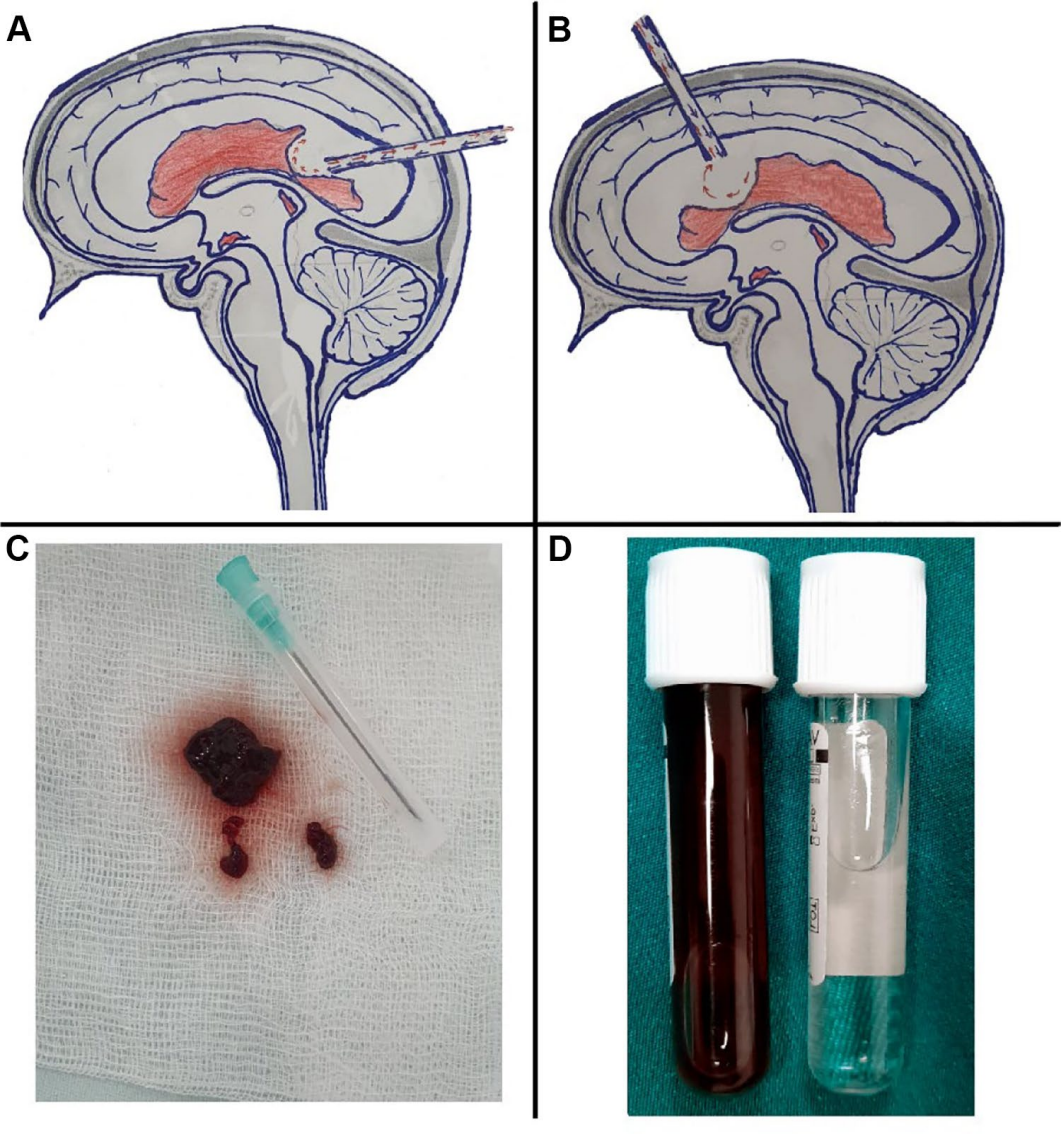
**A**, **B** Schematic drawing of endoscopic trajectory of the occipital and frontal horn of the lateral ventricle. **C** Blood clots removed by NEL. **D** CSF before and immediately after NEL

In the endoscopic group (A), at the first stage, all patients underwent endoscopic intervention—endoscopic lavage and irrigation of the ventricular system as well as the evacuation of posthemorrhagic debris. A surgical approach was performed in 2 and 17 patients from the anterior horn of the lateral ventricle (Kocher’s point) and posterior horn of the lateral ventricle (Dandy’s point), respectively. A neuroendoscopy system was introduced in the frontal or occipital horn of the lateral ventricle with a greater amount of blood. After insertion of the endoscope into the ventricle, continuous irrigation with Ringer’s solution was performed through the endoscope irrigation cannula (Ringer’s temperature was up to 35–37 °C). In two cases, septostomy and irrigation of the second lateral ventricle were also performed, but the results were unsatisfactory (equal cleaning of both lateral ventricles could not be achieved). Irrigation was continued until blood residues were washed out of the ventricular system. For irrigation, we used Ringer’s solution in the amount of 2000–2500 ml (Fig. 1). Upon completion of irrigation, the ventricular reservoir was placed through the existing post-endoscopic canal. All patients were treated with a single neuroendoscopic lavage. Postoperatively, preventive antibiotic therapy was administered to all patients. Ultrasound control of head circumference and ventricular dilatation were performed routinely 2 times a week. If progressive ventricular dilation with signs of hydrocephalus was observed in the postoperative period, subcutaneous ventricular reservoir puncture with excess fluid evacuation was carried out.

Shunting: If active hydrocephalus persisted despite treatment/punctures, the patient was prepared for further surgical treatment such as ventriculoperitoneal shunting. The shunting criterion was standard. At the time of shunting, all patients weighed more than 1600 g.

In the traditional group (B), 24 neonates underwent ventricular reservoir implantation and 12 neonates underwent ventriculostomy, respectively. Surgical treatment indications and postoperative management were similar to those in group A. Indications for shunting were similar in both groups.

Because of their poor clinical condition, 5 newborns were delayed for their first temporary surgical intervention and received only serial ventricular/lumbar punctures until VP shunting (group C).

After discharge from the clinic, 15 patients from group A, 21 patients from group B, and 2 patients from group C were tested by the Denver Developmental Screening Test in the 6th and 12th months of their life.

## Results

In group A, the average gestational age was 31 weeks with an average birth weight of 1423 g. The group comprised 10 female and 9 male patients. Moreover, the average number of neurosurgical interventions in group A was 2.73 (range 1–4). In this group, 3 (15.78%), 14 (73.68%), and 2 (10.52%) neonates were diagnosed with grade 2, grade 3, and grade 4 intraventricular hemorrhages, respectively. The average age in the group at the first neurosurgical procedure was 22 days (range 5–35 days). In group A, 15 neonates (78.94%) underwent shunting during the hospital stay, and 4 patients (21.05%) were discharged from the clinic without any need for a fluid drainage device. Three patients (15.78%) in group A developed meningitis. At final neurosonoscopy, focal white matter injury was reported in 8 patients (42.10%), and 1 (5.26%) patient developed multiloculated hydrocephalus. The average length of hospital stay in group A was 126 days (Table 1).

**Table 1:** Patient characteristics

**Variable**	**Group A**	**Group B**	**Group B** **VAD cohort**	**Group B** **EVD cohort**	**Group C**
No. of patients	19	36	24	12	5
Median gestation age at birth (wks)	31	29.51	30	27.1	29
Median weight at birth (g)	1423	1391	1515	1550	1205
IVH Grade					
I Grade	0	0	0	0	0
II Grade	3 (15.78%)	$8 (22.22\mathrm{ \%})$	6 (25%)	2(16.6%)	$1 (20\mathrm{\%})$
III Grade	14 (73.68%)	$24 (66.66\mathrm{\%})$	17 (70.8%)	7(58.3%)	$3 (60\%)$
IV Grade	2 (10.52%)	$4 (11,11\mathrm{\%})$	1 (4.1%)	$3 (25\%)$	$1 (20\%)$
Sex					
Male %	9 (47.36%)	17(52.7%)	13	4	2(40%)
Female %	10 (52.63%)	19(47.2%)	11	8	3(60%)

In group B, a total of 36 patients with an average gestational age of 29.51 weeks and an average birth weight of 1391 g were included. In the group, 24 neonates underwent ventricular reservoir implantation (VAD), and 12 neonates had EVD. The average number of neurosurgical interventions in the group was 2.86 (range 1–11), while among survivors, the number amounted to 3 (range 1–11).

In group B, 8 (22.22%), 24 (66.66%), and 4 (11.11%) neonates were diagnosed with grade 2, grade 3, and grade 4 intraventricular hemorrhages, respectively. On average, the first surgery was performed on the 37th day after birth (5–158 days). For the VAD cohort, it was carried out 42 days after birth and for the EVD cohort, 28 days after. During hospitalization for patients in group B, 26 (83.87%) neonates required shunting, and 5 patients died before the need for shunting was identified. Meanwhile, 5 patients (13.88%) left the clinic without undergoing shunting. In the group, 18 patients (50%) developed meningitis, of which 10 neonates were from the VAD cohort and 8 from the EVD cohort. Regarding white matter injury, at final neurosonoscopy, the minimal focal injury was reported in 11 (30.5%) patients of which 4 neonates (16.6%) were from the VAD cohort, and 7 (58.3%) were from the EVD cohort. Eleven (30.5%) patients developed multiloculated hydrocephalus, and 9 patients died (25%). The average length of hospitalization for patients in group B was 134 days, with survivors averaging 152 days. The average length of hospitalization for the VAD cohort was 138 days, and for the EVD cohort, it was 128 days (Table 2).

**Table 2:** Treatment outcomes

Variable	Group A	Group B	*P*-value	Group B VAD cohort	Group B EVD cohort	Group C
Multiloculated hydrocephalus%	1 (5.26%)	$11$(30.55%)	^*p* < 0.05^	4 (16.6%)	$7 (58.3\%)$	4(80%)
VP Shunt rate %	15 (78.94%)	$26 (83.87\mathrm{\%})$	^*p* > 0.05^	18 (81.81)	$8 (80\%)$	Not valid*
Mortality rate %	$0$	$9 (25\%)$	^*p* < 0.05^	6 (25%)	$3$(25%)	Not valid*
Median rate of neurosurgical intervention	$2.73$	$2.86$		2.43	$3.72$	1.4

* For group C, because of specific criteria (which are described in the manuscript), these data are not reliable

In group C, the average gestational age was 29 weeks with an average birth weight of 1205 g. In this group, 1 (20%), 3 (60%), and 1 (20%) neonate was diagnosed with grade 2, grade 3, and grade 4 intraventricular hemorrhages, respectively. The first surgery was performed on the 55th day after birth on average (42–75 days). Regarding white matter injury, at the final neurosonoscopy, all patients showed some kind of brain parenchymal damage. Four (80%) patients developed multiloculated hydrocephalus. Three (40%) of the 5 patients died within the 1st year, and none of these deaths were due related to postoperative complications of the procedure. Instead, they were related to respiratory, cardiac, and infectious conditions associated with premature birth. A 12-month neurological evaluation was completed in only 2 cases, and both of them had a severe motor and cognitive neurological disorders.

### Neurodevelopmental outcome

At 6 and 12 months after birth, 15 patients from group A and 21 patients from group B were tested for their neurological outcomes. From group A, 9 (60%) patients did not have any neurological disorder, 4 (26.6%) had a mild motor dysfunction (spastic diplegia/diparesis and monoparesis), and 2 (13.3%) exhibited symptoms of mixed-type cerebral palsy. Furthermore, neurocognitive disorders were observed in 6 cases, of which 3 patients had a mild neurocognitive disability and 3 had poorer cognitive results.

In group B, 5 (23.8%) had a mild motor dysfunction (spastic diplegia/diparesis and monoparesis), and 10 (47.6%) showed symptoms of mixed-type cerebral palsy. Neurocognitive disorders were also observed in 15 cases, of which 4 patients had mild neurocognitive disabilities and 11 had poorer cognitive results (Table 3).

**Table 3:** Neurological outcomes

By Denver Developmental Screening	Group A	Group B
No. of patients	$15$	$21$
No neurological disorder	9 (60%)	$6 (28.5\mathrm{\%})$
Motor dysfunction	6 (40%)	15 (71.4%)
Mild	4 (26.6%)	5 (23.8%)
Severe	2 (13.3%)	$10 (47.6\mathrm{\%})$
Neurocognitive disorder	6 (40%)	$15 (71.4\%)$
Mild	3 (20%)	$4 (19\%)$
Severe	3 (20%)	11 (52.3%)

## Discussion

Intraventricular hemorrhage (IVH) is a common and clinically significant problem in preterm infants. A large IVH is often complicated by posthemorrhagic ventricular dilation [8, 10]. This is especially the case in preterm infants with a large IVH. Because the onset of posthemorrhagic hydrocephalus starts with the initial hemorrhage [11], the removal of the intraventricular hematoma, which is the causative agent, may be a possible treatment option. This procedure has been established for the treatment of adult IVH.

Intraventricular fibrinolytic therapy with streptokinase cannot be recommended for neonates following IVH because of the increased risks of rehemorrhage and failure to reduce shunt dependency within the treatment group [11]. Previous studies also demonstrated that agents like acetazolamide and furosemide did not decrease the need for permanent shunt insertion or the likelihood of mortality and neurological morbidity. Moreover, pharmacotherapy is not recommended for the management of PHH [12].

External ventricular drainage is one of the safe techniques that can be used for the initial treatment of PHH, and it is a reliable first-line treatment for posthemorrhagic hydrocephalus [13, 14]. However, external ventricular drainage can be associated with infection rates up to 50%. In addition, the less developed the mechanisms for the clearance of cerebrospinal fluid (CSF), the greater the risk of requiring permanent cerebrospinal fluid diversion [2].

Ventricular reservoirs are the most common alternative technique for the treatment of IVH/PHH in infants. VADs are simple reservoirs implanted underneath the skin and directly connected to a ventricular catheter to drain excess CSF and reduce intracranial pressure (ICP). VADs are less traumatic than serial lumbar or ventricular punctures. Despite their usefulness in the management of PHH, VAD use can be associated with a number of complications such as local wound/skin problems, CSF leak, catheter migration, and infection [2].

In their fundamental work, Schulz et al. demonstrate the endoscopic irrigation of the ventricular system using Ringer’s solution for the treatment of IVH/PHH in infants. Advantages of this method over the method of irrigation by fibrinolytic drugs include the following: (1) much shorter duration of intervention and (2) the use of Ringer’s solution, which rules out additional fibrinolytic activity. Therefore, the risk of repeated hemorrhage is minimal.

In our findings, NEL decreases the incidence of meningitis and the frequency of developing multiloculated hydrocephalus. It also reduces the period of hospital stay (Table 3). In group A, shunt survival at 1 year since birth was 60%, and it was 45% in group B. In the NEL group, we achieved better motor and neurocognitive outcomes. We suppose that the presentation of multifocal hydrocephalus or the presence of any other deep parenchymal damage, including grade 4 hemorrhage, could be regarded as the most predisposing factor for the development of deep neurological disorders. In our study, the average duration of neuroendoscopic surgery was 55 min, which can be considered a relative disadvantage of this method. Based on severe general comorbidities and considering equal brain damage, traditional methods were chosen as treatment procedures in most cases (taking into account the shorter duration of surgery), which is reflected in the high rate of lethal outcomes in group B.

Our data support the findings of recent studies published in the literature. Schulz and Etus retrospectively compared the complications and potential benefits for neonates who have been treated with neuroendoscopic ventricular lavage for their PHH. According to their results, endoscopic ventricular lavage was associated with fewer overall necessary procedures, significantly fewer infections, and multiloculated hydrocephalus development [2, 4, 15, 16]. Frassanito et al. compared the results of initial ventriculosubgaleal shunt (VSgS) with the NEL–VSgS combination. They concluded that VSgS and NEL are two effective treatment options. Both procedures should be part of the neurosurgical armamentarium to deal with PHH [17]. Honeyman et al. confirmed that NEL is a safe and potentially efficacious treatment for neonatal IVH [18]. The procedure may reduce shunt dependence and improve shunt survival for those who require CSF diversion [3].

### Limitations of the study

Due to the peculiarities of the Georgian healthcare system, it is not possible to adequately monitor all patients after discharge from the clinic, which limits our ability to accurately record the final neurological outcomes.

The general condition of patients in the NEL group (i.e. comorbidities) was relatively mild, ultimately reflected in the frequency of lethal outcomes.

In most cases, we used a different form of NEL from that described in the literature. We used a single lateral ventricle approach (only 2 cases out of 19 used septostomy and irrigation of the second lateral ventricle). This may partly hinder the effects of this procedure on the reduction of shunt dependency. In addition, the 2 cases where we used septostomy and irrigation of the second lateral ventricle VP shunting were performed because of the progression of hydrocephalus.

Notwithstanding the above, we believe that the results of the study are credible. Nonetheless, a broader multicenter study is needed for a more comprehensive exploration of this issue.

## Conclusion

Neuroendoscopic lavage is a safe and effective method of treatment. Its use is not associated with an increased risk of developing repeated hemorrhage. No intraoperative or postoperative complications that are directly associated with the application of the neuroendoscopic method were observed at any stage of the study.
